# Birth Cohort Differences in Multimorbidity Burden Among Aging U.S. Adults

**DOI:** 10.1093/geroni/igab046.987

**Published:** 2021-12-17

**Authors:** Nicholas Bishop, Steven Haas, Ana Quiñones

**Affiliations:** 1 Texas State University San Marcos, San Marcos, Texas, United States; 2 Penn State, University Park, Pennsylvania, United States; 3 Oregon Health & Science University, Portland, Oregon, United States

## Abstract

Multimorbidity is the co-occurrence of two or more chronic health conditions and affects more than half of the US population aged 65 and older. Recent trends suggest increased risk of poor self-reported health, physical disability, cognitive impairment, and mortality among later born birth cohorts, yet we are unaware of work examining cohort trends in multimorbidity among aging US adults. Observations were drawn from the Health and Retirement Study (2000–2018) and included adults aged 51 and older across 7 birth cohorts (1923 and earlier, 1924–1930, 1931–1941, 1942–1947, 1948–1953, 1954–1959, and 1960–1965). Multimorbidity was measured as a count of 9 chronic conditions including heart disease, hypertension, stroke, diabetes, arthritis, lung disease, cancer (excluding skin cancer), depression, and cognitive impairment. General linear models adjusting for repeated measures and covariates including age, sex, race/ethnicity, and education were used to identify whether trends in multimorbidity varied across birth cohort. 31,923 adults contributed 153,940 total observations, grand mean age was 68.0 (SD=10.09), and mean multimorbidity was 2.19 (SD=1.49). In analyses adjusted for age and other covariates, adults born 1948–1953 reported .34 more chronic conditions (SE=.03, p<.001), adults born 1954–1959 reported .42 more chronic conditions (SE=.03, p<.001), and adults born 1960–1965 reported .55 more chronic conditions (SE=.03, p<.001), than those born 1931–1941, respectively. Our preliminary results confirm increasing multimorbidity among later birth cohorts of older Americans and should help guide policy to manage impending health declines among older Americans.

